# Issues for application of virtual microscopy to cytoscreening, perspectives based on questionnaire to Japanese cytotechnologists

**DOI:** 10.1186/1746-1596-3-S1-S15

**Published:** 2008-07-15

**Authors:** Ichiro Mori, Osamu Nunobiki, Takashi Ozaki, Emiko Taniguchi, Kennichi Kakudo

**Affiliations:** 1 Department of Pathology, School of Medicine, Wakayama Medical University. 811-1 Kimiidera, Wakayama, 641-8509, Japan; 2 Faculty of Health Sciences, Kobe Tokiwa University. 2-6-2 Ohtanichou, Nagataku, Kobe, 653-0838, Japan

## Abstract

To clarify the issues associated with the applications of virtual microscopy to the daily cytology slide screening, we conducted a survey at a slide conference of cytology. The survey was conducted specifically to the Japanese cytology technologists who use microscopes on a routine basis. Virtual slides (VS) were prepared from cytology slides using NanoZoomer (Hamamatsu Photonics, Japan), which is capable of adjusting focus on any part of the slide. A total of ten layers were scanned from the same slides, with 2 micrometer intervals. To simulate the cytology slide screening, no marker points were created. The total data volume of six slides was approximately 25 Giga Bytes. The slides were stored on the Windows 2003 Server, and were made accessible on the web to the cytology technologists. Most cytotechnologists answered "Satisfied" or "Acceptable" to the VS resolution and drawing speed, and "Dissatisfied" to the operation speed. To the ten layered focus, an answer "insufficient" was slightly more frequent than the answer "sufficient", while no one answered "fewer is acceptable" or "no need for depth". As for the use of cytology slide screening, answers "usable, but requires effort" and "not usable" were about equal in number. In a Japanese cytology meeting, a unique VS system has been used in slide conferences with markings to the discussion point for years. Therefore, Japanese cytotechnologists are relatively well accustomed to the use of VS, and the survey results showed that they regarded VS more positively than we expected. Currently, VS has the acceptable resolution and drawing speed even on the web. Most cytotechnologists regard the focusing capability crucial for cytology slide screening, but the consequential enlargement of data size, longer scanning time, and slower drawing speed are the issues that are yet to be resolved.

## Introduction

In April 2007, Japanese government introduced approximately 100 Virtual Slide (VS) scanning machines to various medical facilities. We are currently accumulating various ideas and experiences for the applications of the VS including routine pathology diagnosis [1]. These ideas are usually sought among the doctors of pathology. However, since there are many cytotechnologists in Japan who also use microscopes on a daily basis, we have conducted a survey on the cytotechnologists at a cytology conference. To imitate the conditions of the cytology slide screening, we prepared VSs that had no marker point set and were capable of adjusting the focus in any part of the slide.

## Materials and methods

We prepared VS data using NanoZoomer (Hamamatsu Photonics, Japan) that has Z-stack scan capability that allows the focus to move three dimensionally to any part of the slide [2,3]. We used a 40× objective lens to scan 6 slides. Each VS was composed of 10 layers with 2 micrometer intervals. We scanned the slides overnight using an automatic slide loader. The scanned areas varied from 15.9 × 12.7 mm to 44.0 × 35.1 mm depending on the specimen dubbed areas on the slide glasses. The VS data were stored on the Windows 2003 Server of Wakayama Regional Medical Information Network Association so that they can be accessible to the cytology technologists. Free downloadable software for NanoZoomer was also provided on the same server. The VS operational manual, IP address of the server, access ID and password were printed in the meeting program booklet and sent to the cytologists by mail along with the questionnaire.

## Results

According to Hamamatsu Photonics, given a scanning area of 20 × 20 mm and an objective lens of 20×, Nanozoomer can scan one slide in 3 minutes [2]. Because the cytology slides usually require more detailed observation than the histology slides, we adjusted the scan such that the VSs have greater magnification and focusing ability. The largest slide glass took about 4 hours to scan and the data size was 154 times more than the typical scan, due to larger area (44.0 × 35.1 mm), greater magnification (40× lens), and 10 Z-stack planes. The data size was 9.7 Giga Bytes (GB) after compression (520 GB before compression). The smallest slide took 33 minutes to scan and the data size was 1.11 GB. Total data size of the 6 slides was approximately 25 GB.

After overnight automatic scan of the 6 slides, we found a scanning error in one of the slides, and an additional manual scan was performed on the slide.

A total of 164 cytotechnologists attended the conference, 62 answers were retrieved. Of those 62, 22 did not have a look at the VS, and 10 answered that they were unable to connect to the server. Of the 39 that actually looked at the VS on the web, a majority had the internet connection environment of workplace LAN (30/39), while 6 had FTTH (optical Fiber to the Home), and one cytologist had ADSL. For reference purpose, I personally checked connection speeds of LAN at my workplace and of FTTH at my home. The LAN had the connection speed of 5 to 8 Mbps and FTTH had 18 to 20 Mbps. From experience, I felt the difference between LAN and FTTH was not significant.

**Figure 1:**
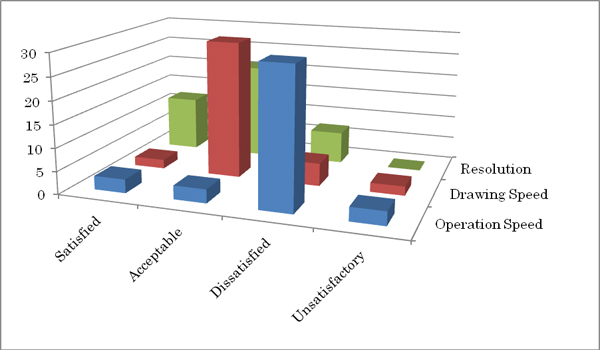
Satisfaction level. Relatively high in resolution but low in operation speed.

**Figure 2:**
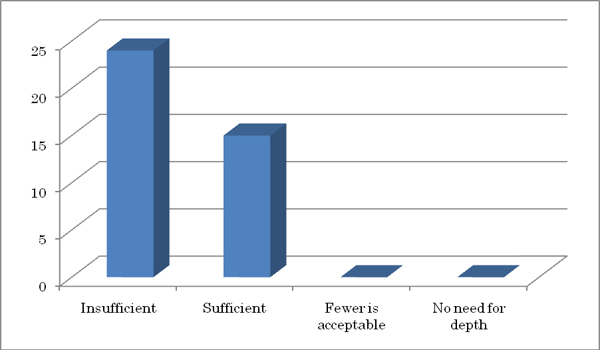
Sufficiency of the ten layered focus. An answer "insufficient" was slightly more frequent than the answer "sufficient".

Of the 39 cytologists that have looked at the VS, 12 gave an answer "Satisfied" to the resolution of the VS, while 21 gave "Acceptable," 7 gave "Dissatisfied," and no one gave "Unsatisfactory". In regard to the drawing speed, 2 answered "Satisfied," 30 gave "Acceptable," 5 gave "Dissatisfied," and 2 gave "unsatisfactory." In regard to the operation speed, 3 gave "Satisfied," 3 gave "Acceptable," 30 gave "Dissatisfied," and 3 gave "Unsatisfactory" (Figure 1). On the 10-layered focus ability, 24 answered "insufficient" and 15 answered "sufficient," while no one answered, "fewer is acceptable" or "no need for depth" (Figure 2).

**Figure 3:**
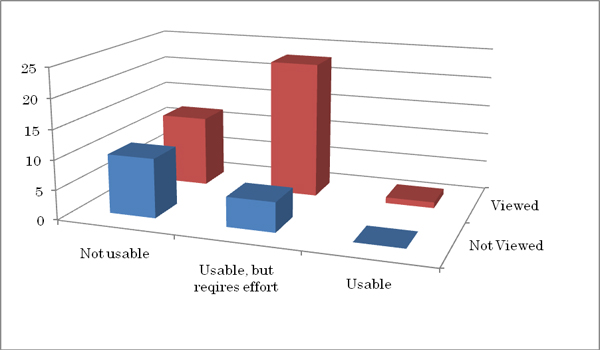
Usability for cytology slide screening. More cytotechnologist who actually viewed the VS of NanoZoomer answered "usable, but requires effort" while who didn't viewed answered "not usable".

For the use of VS to the cytology slide screening, 28 answered, "Usable, but requires effort," while 22 answered "Not usable." Among the 28 that answered, "Usable, but requires effort," 23 had looked at the VS, whilst 5 had not. For the 22 that answered "Not usable," 12 of the cytotechnologists had looked at the VS, and 10 had not (Figure 3).

## Discussion

In past meetings of the Japanese Society of Clinical Cytology, a unique VS system [4], developed by a member of the Society, had been used for years. This system puts markings to the discussion point and has ability to change the focus, though limited to the discussion point. Therefore, Japanese cytologists are relatively accustomed users of the VS. In addition, manufacturers at each conference actively perform VS system demonstrations, and some cytotechnologists may have already tried several different VS systems. Perhaps because of these reasons, the survey results showed that the cytologists regarded VS more positively than we originally expected.

The NanoZoomer, the VS system we used for this survey, has Z-stack function that allows the focus to move three dimensionally to any part of the slide [2], and perhaps a step ahead of the other VS systems. On the other hand, the added depth of the scan led to a new issue of increase in scanning time.

The large volume of the VS data, 25 GB/6 slides in our case, is another issue with NanoZoomer. We had to use removable hard disks to transport the data of only 6 slides. If such VS data is used in routine diagnosis, they will quickly fill up the server storage space. Larger data size also leads to slower communication. When we looked at the VS data on the local computer, we felt no problems with drawing speed and handling speed. On the contrary, when the same VS is looked at through the Internet, we felt the response to be slow and frustrating. One of the issues with the slow response is the difficulty of finding the appropriate focus. The viewer of the NanoZoomer changes focus using the mouse wheel. When the response is quick, there was no problem in finding the appropriate focus. However, when the response gets slow, we often missed the right focus, and some of us even felt motion sickness due to the repeated focus adjustment.

The NanoZoomer has a slide loader that can load up to 210 slides. When the slides are such that the NanoZoomer can scan each slide in 3 minutes, all the 210 slides can be converted into VS by overnight scan, and can be fitted in daily diagnosis schedule easily. However, in our case, scanning the largest slide took 4 hours. In addition, the slide loader failed to scan on one of the slides in the automatic overnight scan. According to the Hamamatsu Photonics, such failure may occur if the staining is too faint, specimen is too small, or slide glass dirty.

In regard to the applicability of the VS to daily cytology slide screening, those who actually looked at the VS answered more positively to the questionnaire than those who did not. The ratio of the answers "Usable, but requires effort" versus "Not usable" were 23:12 for those that looked at the VS, while the ratio was 5:10 for those that did not look at the VS. The most significant difference between VS of NanoZoomer and other system is the flexibility of the focus in examining the VS, and this difference might have influenced their opinion.

## Conclusion

As a conclusion, we determined that the Japanese cytologists consider the focusing capability crucial for cytology slide screening. However, as a result it led to other issues such as larger data size, longer scanning time, and slower drawing speed that still need to be resolved for the VS to become fully accepted. These issues become especially important when the data is to be transferred through the Internet, for these issues make the communication slow and unstable. Under current conditions, VS remains "Usable, but requires effort" for cytology slide screening even by those who regard the VS rather positively.
